# Autotetraploid plant regeneration by indirect somatic embryogenesis from leaf mesophyll protoplasts of diploid *Gentiana decumbens* L.f.

**DOI:** 10.1007/s11627-015-9674-0

**Published:** 2015-03-11

**Authors:** Karolina Tomiczak, Anna Mikuła, Elwira Sliwinska, Jan J. Rybczyński

**Affiliations:** 1 Department of Experimental Plant Biology, Polish Academy of Sciences Botanical Garden–Center for Biological Diversity in Powsin, Prawdziwka 2, 02-973 Warsaw, Poland; 2 Department of Plant Genetics, Physiology and Biotechnology, Laboratory of Molecular Biology and Cytometry, University of Technology and Life Sciences, Kaliskiego Ave. 7, 85-789 Bydgoszcz, Poland

**Keywords:** Protoplast culture, Somaclonal variation, Gentian, Flow cytometry, Chromosome number

## Abstract

Somaclonal variation, often manifested as the increased ploidy of plants observed following *in vitro* culture, can be advantageous in ornamental species or those used for secondary metabolite production. Polyploidy occurs especially when plantlets are produced by protoplast and callus cultures. Plants were regenerated from green leaf mesophyll protoplasts of diploid *Gentiana decumbens* L.f. through somatic embryogenesis. A yield of more than 9 × 10^5^ protoplasts per gram of fresh weight was achieved by incubating fully expanded young leaves in an enzyme mixture containing 1.0% (*w/v*) cellulase and 0.5% (*w/v*) macerozyme. Protoplasts, cultured in agarose beads using a modified Murashige and Skoog medium, divided and formed microcalli, with the highest plating efficiency obtained on medium containing 2.0 mg l^−1^ 1-naphthaleneacetic acid and 0.1 mg l^−1^ thidiazuron. Callus proliferation was also promoted by including thidiazuron in agar-solidified medium, while somatic embryogenesis was induced from microcalli on medium supplemented with 1.0 mg l^−1^ kinetin, 0.5 mg l^−1^ gibberellic acid, and 80 mg l^−1^ adenine sulfate. Flow cytometric analysis and chromosome counting revealed that all regenerants were tetraploid.

## Introduction

*Gentiana decumbens* L.f., is one of about 360 *Gentiana* species, well known for their pharmacological properties and decorative flowers. It is a diploid (2*n* = 2*x* = 26) perennial herb growing on grassland slopes and dry steppes of Kazakhstan, Mongolia, Russia, and China (Ho and Pringle 1995). According to the classification of Ho and Liu (1990), this species, together with *Gentiana crassicaulis* Duthie ex Burk., *Gentiana cruciata* L., *Gentiana kurroo* Royle, *Gentiana macrophylla* Pall., *Gentiana straminea* Maxim*.*, and *Gentiana tibetica* King, belongs taxonomically to the section Cruciata, comprising diploid and tetraploid species with 26 or 52 chromosomes, respectively. *G. decumbens*, like other gentians, is a source of bitter secoiridoid glucosides and flavonoids (Dungerdorj *et al.* 2006), and it possesses high antioxidant potency both *in vitro* and *ex vivo* (Myagmar and Aniya 2000). *G. decumbens* is used extensively in traditional Mongolian medicine for the treatment of liver diseases (Dungerdorj *et al.* 2006; Kletter *et al.* 2008), while in Pakistan, a tincture of this plant is prepared to treat stomach disorders (Qureshi *et al.* 2007). Due to its blue flowers and ease of cultivation, *G. decumbens* is also planted in rock and alpine gardens (Köhlein 1991).

Modern biotechnology offers important tools for mass propagation and genetic improvement of medicinal and ornamental plants. In the genus *Gentiana*, protocols for efficient *in vitro* multiplication have been described for at least 18 species, initiated from a wide range of explant types (Rybczyński *et al.* 2015). The high morphogenic potential of most of these species facilitates the *in vitro* genetic manipulations of these plants, carried out mainly *via Agrobacterium*-mediated transformation (Rybczyński *et al.* 2015). Protoplasts could also be used for plant genetic improvement by somatic hybridization, direct genetic transformation, and somaclonal variation. These approaches could be implemented to produce novel *Gentiana* genotypes and hybrids with desirable secondary metabolite content or visually attractive phenotypes (Nishihara *et al.* 2003). However, the use of protoplast technology for these reasons requires the development of effective protocols for plant regeneration from cultured protoplasts.

To date, four *Gentiana* species and one interspecific hybrid have been regenerated from protoplasts. Takahata and Jomori (1989) first reported low-frequency shoot organogenesis from green leaf mesophyll protoplasts of *Gentiana scabra* Bunge. Plant regeneration from mesophyll protoplasts of *Gentiana triflora* Pall. and *Gentiana triflora* × *Gentiana scabra via* organogenesis have also been reported (Nakano *et al.* 1995). In contrast, use of somatic embryogenesis as the route for plant regeneration from protoplasts has been restricted so far to protoplasts derived from undifferentiated plant material such as embryogenic callus of *G. crassicaulis*, induced from hypocotyl explants (Meng *et al.* 1996), or from highly embryogenic cell suspensions of *G. kurroo* (Fiuk and Rybczyński 2007).

The aim of this work was to develop a protocol for efficient plant regeneration from protoplasts of differentiated green leaf mesophyll cells of *G. decumbens*. The regenerants were evaluated using both direct and indirect methods to estimate ploidy.

## Materials and Methods

### Plant Material.

Seeds of *G. decumbens* were obtained from the Berlin-Dahlem Botanical Garden and Botanical Museum in Berlin, Germany. The seeds were surface sterilized for 30 s in 70% (*v/v*) ethanol, followed by 15 min in 1% (*v/v*) sodium hypochlorite solution, followed by three rinses in sterile water. Seeds were placed on half-strength Murashige and Skoog (MS) medium (Murashige and Skoog 1962) supplemented with 0.5 mg l^−1^ gibberellic acid (GA_3_), 10 g l^−1^ sucrose, and 7 g l^−1^ agar. The pH of the medium was adjusted to 5.8 with 0.1 M NaOH or 0.1 M HCl before autoclaving at 121°C for 18 min. After 3 wk, seedlings were transferred to 450-ml glass jars containing 80 ml of half-strength MS medium with 15 g l^−1^ sucrose and 8 g l^−1^ agar, and maintained in a growth chamber at 21 ± 1°C with 16-h illumination of 150 μM m^−2^ s^−1^ provided by daylight fluorescent tubes. The plantlets were subcultured to new medium every 3–4 mo.

### Protoplast Isolation.

Fully expanded, young leaves were harvested from *in vitro*-grown *G. decumbens* plants. After removal of the lower epidermis, 1 g of leaf material was submerged in 10 ml of cell and protoplast wash solution (CPW; Frearson *et al.* 1973) supplemented with 0.33 M mannitol (pH 5.8) for 30 min of pre-plasmolysis. Leaf tissues were then incubated in filter-sterilized enzyme solution for 3–4 h in the dark at 26°C on a rotary shaker (50 rpm). The enzyme mixture consisted of 1.0% (*w/v*) Cellulase Onozuka R-10 and 0.5% (*w/v*) Macerozyme R-10 (both from Yakult Honsha Co., Ltd., Tokyo, Japan), dissolved in CPW solution with 0.5 M mannitol and 5 mM 2-(*N*-morpholino)ethanesulfonic acid (MES), pH 5.8. Released protoplasts were filtered through a 45-μm nylon sieve and collected by centrifugation at 180 rpm for 10 min. The pellet was resuspended in CPW solution containing 0.5 M mannitol and centrifuged at 180 rpm for 10 min. This centrifugation and resuspension procedure was performed three times.

### Microscopic Evaluation of Protoplasts.

For observation of protoplasts, an epifluorescence microscope (Vanox AHBT3; Olympus, Tokyo, Japan) was used. The yield of freshly isolated protoplasts was determined using a Bürker counting chamber and expressed as the number of protoplasts obtained from 1 g of fresh weight of leaves. Protoplast diameters were measured using analySIS® FIVE software (Olympus). Viability was estimated by staining protoplasts with 0.01% (*w/v*) fluorescein diacetate (FDA) solution (Larkin 1976). The mean yield, diameter, and viability of protoplasts were calculated on the basis of data from 6 independent isolations, and 10 microscopic fields of view per isolation.

### Protoplast Culture.

Purified protoplasts were suspended at a density of 1.0 × 10^5^ cells per milliliter in protoplast culture medium (PCM; Table 1) supplemented with 0.5 M mannitol and 0.8% (*w/v*) Sea Plaque® Agarose (Cambrex Bio Science Rockland Inc., Rockland, ME). Twelve to 14 agarose beads each of 100 μl in volume and about 7 mm in diameter were formed on each 50-mm Petri dish. After setting, the beads were submerged in 5.0 ml of PCM liquid medium.

**Table 1.** Media used for the culture of *Gentiana* protoplasts, callus proliferation, and plant regeneration

Medium code	Medium components
PCM1	MS salts (without NH_4_NO_3_) and vitamins, 30 g l^−1^ glucose, 3.0 g l^−1^ glutamine, 2.0 mg l^−1^ NAA, 0.1 mg l^−1^ TDZ^a^
PCM2	MS salts (without NH_4_NO_3_) and vitamins, 30 g l^−1^ glucose, 3.0 g l^−1^ glutamine, 2.0 mg l^−1^ NAA, 1.0 mg l^−1^ BAP^a^
CPM1	MS salts and vitamins, 30 g l^−1^ sucrose, 2.0 mg l^−1^ NAA, 0.2 mg l^−1^ TDZ
CPM2	MS salts and vitamins, 30 g l^−1^ sucrose, 2.0 mg l^−1^ NAA, 1.0 mg l^−1^ BAP
CPM3	MS salts and vitamins, 30 g l^−1^ sucrose, 1.0 mg l^−1^ dicamba, 0.1 mg/l^−1^ NAA, 2.0 mg l^−1^ BAP, 80 mg l^−1^ adenine sulfate
CPM4	MS salts and vitamins, 30 g l^−1^ sucrose, 0.5 mg l^−1^ 2,4-D, 1.0 mg l^−1^ kinetin
PRM1	MS salts and vitamins, 30 g l^−1^ sucrose, 20 ml l^−1^ coconut water, 0.1 mg l^−1^ NAA, 8.0 mg l^−1^ TDZ
PRM2	MS salts and vitamins, 30 g l^−1^ sucrose, 20 ml l^−1^ coconut water, 0.1 mg l^−1^ NAA, 6.0 mg l^−1^ BAP
PRM3	MS salts and vitamins, 30 g l^−1^ sucrose, 20 ml l^−1^ coconut water, 1.0 mg l^−1^ kinetin, 0.5 mg l^−1^ GA_3_, 80 mg l^−1^ adenine sulfate
PRM4	MS salts and vitamins, 30 g l^−1^ sucrose, 20 ml l^−1^ coconut water, 1.0 mg l^−1^ NAA, 2.0 mg l^−1^ BAP, 3.0 mg l^−1^ zeatin, 1.0 mg l^−1^ GA_3_

*PCM* protoplast culture medium, *CPM* callus proliferation medium, *PRM* plant regeneration medium

^a^PCM media were supplemented with different concentrations of mannitol (0.5, 0.33, 0.17, or 0.0 M), as described in the “Materials and Methods”

Protoplast cultures were incubated at either 21 or 26°C in the dark. During the first week of culture, cell wall regeneration was monitored by staining protoplasts with 0.001% (*w/v*) calcofluor (Fluorescent Brightener 28, Sigma-Aldrich, St. Louis, MO) solution. On the seventh day of culture, plating efficiency was determined. Dividing cells were counted using an IMT-2 inverted microscope (Olympus) from 6 independent agarose bead cultures, with 10 fields of view per culture. Plating efficiency was expressed as the number of cells that divided at least once per total number of plated protoplasts, multiplied by 100.

To avoid browning of the cultures, 2.5 ml of the liquid medium was replaced with the same amount of the same fresh medium every week. To stimulate further cell division and colony formation, the osmotic pressure of the media was gradually reduced starting from the fifth week of the culture. This was accomplished by replacing 2.5 ml of the current liquid PCM medium with the same amount of medium supplemented with 0.33 M mannitol during the fifth and sixth week of culture, 0.17 M mannitol in the seventh and eighth weeks, and no mannitol in subsequent weeks.

### Callus Culture and Plant Regeneration.

When protoplast-derived microcalli reached about 1–2 mm in diameter, agarose beads were transferred to 90-mm Petri dishes with agar-solidified callus proliferation medium (CPM; Table 1) and cultured in the dark at 26°C. The rate of callus proliferation was estimated 4 wk after transfer to CPM by the comparison of the surface of formed tissue with the surface of the agarose bead. Depending on the proliferation rate, callus lines were placed on agar-solidified plant regeneration medium (PRM) after 4 to 8 wk (Table 1) and cultured under growth chamber conditions (21°C, 16/8 h photoperiod, 150 μM m^−2^ s^−1^ light intensity) to obtain plant regeneration. If no regeneration was observed after 8 wk, callus was transferred onto PRM3 or PRM4 medium for an additional 4 wk. Mature somatic embryos were transferred to glass jars with agar-solidified half-strength MS medium supplemented with 15 g l^−1^ sucrose for germination. Regenerants were subsequently grown on growth-regulator-free MS medium containing 30 g l^−1^ sucrose.

### Flow Cytometry.

Nuclear DNA content was determined for 15 seed-derived plants from which protoplasts were obtained and for 21 independently regenerated, randomly chosen plants. *Pisum sativum* L. ‘Set’ (9.11 pg/2C; Sliwinska *et al.* 2005) served as an internal standard. Young leaves of *G. decumbens* and *P. sativum* were chopped simultaneously using a sharp razor blade in a Petri dish with 1.0 ml of nuclei-isolation buffer (0.1 M Tris, 2.5 mM MgCl_2_ × 6H_2_O, 85 mM NaCl, 0.1% Triton X-100; pH 7.0), supplemented with 50 μg ml^−1^ propidium iodide (PI) and 50 μg ml^−1^ RNase A. After chopping, the suspension was passed through a 50-μm mesh nylon filter. For each sample, at least 7000 nuclei were analyzed using a Partec CCA (Münster, Germany) flow cytometer, equipped with an argon laser. Histograms were analyzed using the DPAC V.2.2 program (Partec GmbH, Münster, Germany). The nuclear DNA content was calculated using the linear relationship between the ratio of the G_0_/G_1_ peak positions of *Gentiana* and *Pisum* on the histogram of fluorescence intensity.

### Chromosome Number Evaluation.

For chromosome counts, roots collected from *in vitro*-grown regenerants and control plants were pretreated with 2 mM 8-hydroxyquinoline at 21°C for 2 h and then at 4°C for 2 h. After fixation in ethanol–acetic acid (3:1, *v/v*), roots were stored at −20°C for at least 24 h. Root tips were hydrolyzed in 5 M HCl for 50 min at 21°C, stained in Schiff’s reagent (Sigma-Aldrich) for 2 h in the dark, rinsed three times with potassium metabisulfite solution (225 mM K_2_S_2_O_5_, 5 mM HCl), squashed in a droplet of 45% (*v/v*) acetic acid on a microscope slide, and observed using a Vanox AHBT3 microscope under ×1000 magnification. The chromosomes were counted using analySIS® FIVE software. At least 10 well-spread metaphase plates were analyzed for each plant.

### Size and Frequency of Stomata.

For stomatal measurements, the youngest pair of fully expanded leaves from *in vitro*-grown plants was used. A fragment of the lower epidermis from the middle portion of the leaf was removed and observed using a Vanox AHBT3 microscope. To determine the guard cell length and stomatal width, all stomata from 10 randomly selected fields of view per each leaf were measured using analySIS® FIVE software. Similarly, all stomata from 10 randomly selected leaf areas per leaf were counted. The stomatal frequency was expressed as a number of stomata per square millimeter of the leaf blade area.

### Statistical Analysis.

Each *in vitro* culture experiment consisted of three replicates. Each replicate comprised three Petri dishes for each combination of culture conditions. One-way analysis of variance (ANOVA) was performed using Statistica 6.0 software (StatSoft Polska Sp. z o.o., Krakow, Poland). Means were compared using Tukey’s honestly significant difference (HSD) test, at the 0.05 level of significance.

## Results and Discussion

### Protoplast Isolation and Culture.

**Figure 1.**
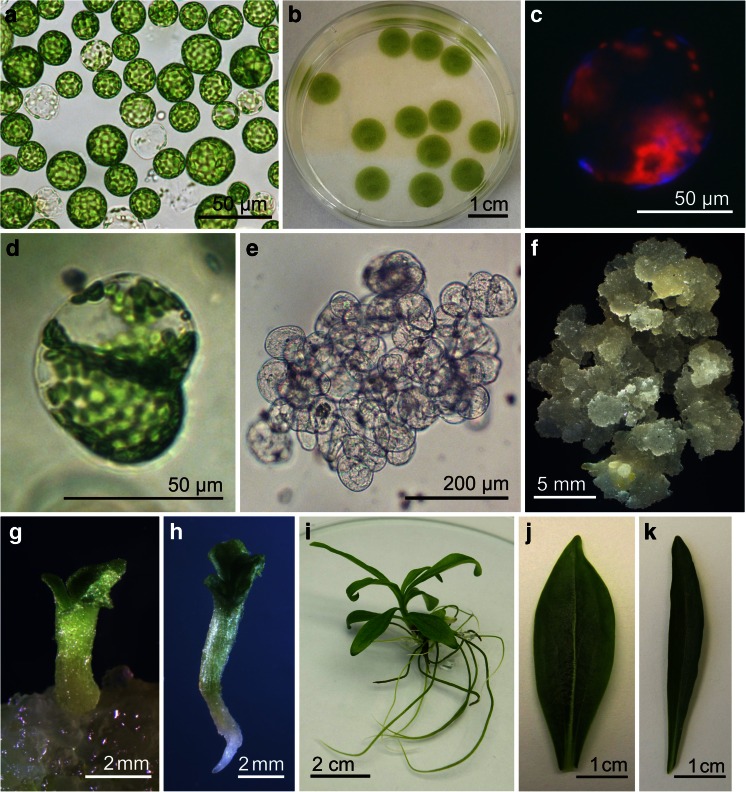
Regeneration of plants from protoplasts of *Gentiana decumbens*: (*a*) freshly isolated green leaf mesophyll protoplasts, (*b*) protoplasts in agarose bead culture, (*c*) regeneration of cell walls demonstrated by calcofluor staining, (*d*) daughter cells formed after the first division of a protoplast-derived cell, (*e*) multicellular aggregate obtained after 7 wk of protoplast culture, (*f*) callus proliferating on CPM4 medium, (*g*) regenerating somatic embryo at the cotyledonary stage, (*h*) mature somatic embryo before conversion into plantlet, (*i*) protoplast-derived plant, (*j*, *k*) leaf morphology of a protoplast-derived regenerant (*j*), and a seed-derived parent plant (*k*).

The development of an efficient protoplast-to-plant system for a species of interest is a prerequisite for further research on its genetic manipulation through somatic hybridization or direct genetic transformation. Although high morphogenic potential has been demonstrated for many *Gentiana* species, expressed either as organogenic (Hosokawa *et al.* 1996; Momčilović *et al.* 1997) or embryogenic regeneration from various explant types (Mikuła and Rybczyński 2001; Fiuk and Rybczyński 2008b; Cai *et al.* 2009), the regeneration of plants from protoplasts has been limited to only a few species and hybrids (Takahata and Jomori 1989; Nakano *et al.* 1995; Meng *et al.* 1996; Fiuk and Rybczyński 2007). We could find no previous reports describing tissue culture of *G. decumbens*; thus, in designing the experiments, reports concerning protoplast culture of *G. scabra*, *G. triflora*, their hybrids, and especially of closely related *G. crassicaulis* and *G. kurroo* were taken into consideration. The enzyme mixture and protoplast isolation protocols described by other researchers working with Gentianaceae (Kunitake *et al.* 1995; Nakano *et al.* 1995) resulted in the release of 9.31 ± 1.30 × 10^5^ protoplasts per gram fresh leaf tissue of *G. decumbens* (Fig. 1*a*). The mean diameter of protoplasts was 24.35 ± 4.94 μm and their viability 84.60 ± 4.08%. Results achieved for *G. decumbens* were similar to those obtained for other gentian species (Nakano *et al.* 1995; Meng *et al.* 1996).

Protoplast culture using agarose beads surrounded by a liquid medium (Fig. 1*b*) was also used successfully by O’Brien and Lindsay (1993) for protoplasts of *Eustoma grandiflorum* Griseb., and its greater efficiency in the induction of cell division in *G. kurroo* compared to liquid and agarose thin-layer cultures was demonstrated by Fiuk and Rybczyński (2007). In agarose droplets, *G. decumbens* mesophyll protoplasts regenerated new cell walls within the first 48 h of culture (Fig. 1*c*) and underwent their first division 3–5 d after isolation (Fig. 1*d*). The second round of divisions started after about 5–7 d of culture.

The extent of cell division depends, among other things, on the media used for protoplast culture and on the physical conditions of incubation (Davey *et al.* 2005). In the present study, a comparison was made of the influence of the two cytokinins added to the protoplast culture medium, *i.e.*, benzylaminopurine (BAP) and thidiazuron (TDZ), on plating efficiency. TDZ at a concentration of 0.1 mg l^−1^ stimulated cell division in cultures of mesophyll protoplasts of *G. triflora* and *G. triflora* × *G. scabra* more than BAP (Nakano *et al.* 1995). Similarly, for *G. decumbens*, a greater percentage of cell divisions was obtained on PCM1 medium, which contained TDZ (Table 2). Also, a temperature of 26°C gave almost twice the plating efficiency obtained at 21°C. However, in spite of differences in rate, cell division continued and multicellular aggregates were obtained within 5–9 wk under all culture conditions tested (Fig. 1*e*). Visible protoplast-derived microcalli formed after 10–12 wk.

**Table 2.** Percentages of dividing leaf mesophyll protoplast-derived cells of *G. decumbens* after 7 d of culture on two different media at two different temperatures

Protoplast culture medium	Temperature of culture incubation (°C)	Percentage of dividing cells (mean ± SD)
PCM1	21	3.11 ± 0.60 b
	26	6.07 ± 2.03 a
PCM2	21	2.15 ± 0.50 b
	26	4.11 ± 0.58 ab

Values followed by the same *letter* are not significantly different at *P* < 0.05 (HSD test)

### Callus Culture and Plant Regeneration.

Callus formation was observed after transfer of microcallus-containing agarose beads onto CPM medium (Fig. 1*f*). Tissue proliferation was far more extensive when protoplasts had been cultured on TDZ-containing medium (PCM1) than on BAP-containing medium (PCM2) (Table 3). Similarly, the largest amount of callus formation was obtained on CPM1 medium, containing 2 mg l^−1^ 1-naphthaleneacetic acid (NAA) and 0.2 mg l^−1^ TDZ. However, another medium effective for callus proliferation was CPM4, containing 2,4-dichlorophenoxyacetic acid (2,4-D) and kinetin (Table 3). This medium was used previously for induction of callus on gentian seedling explants (Mikuła and Rybczyński 2001; Mikuła *et al.* 2002), for the initiation and culture of *G. kurroo* embryogenic cell suspensions (Fiuk and Rybczyński 2008a), and for culture of cell suspension-derived protoplasts (Fiuk and Rybczyński 2007). Tissues proliferating on CPM1 and CPM2 media were creamy colored and well hydrated while granular and yellowish calli formed on CPM3 and CPM4 media. The induction of somatic embryogenesis and the development of the first somatic embryos occurred after 6 wk of culture on CPM3 medium (Fig. 1*g*).

**Table 3.** Effect of protoplast culture medium (PCM) and callus proliferation medium (CPM) on the formation of callus from *G. decumbens* protoplasts after 4 wk of culture

Protoplast culture medium	Callus proliferation medium	Degree of callus formation^a^
PCM1	CPM1	++++
	CPM2	+++
	CPM3	+++
	CPM4	++++
PCM2	CPM1	+
	CPM2	+
	CPM3	+
	CPM4	++

^a^Degree of callus formation is expressed as follows: + very small callus covering not more than a quarter of the agarose bead surface, ++ small callus covering not more than a half of the agarose bead surface, +++ callus not exceeding the surface of the agarose bead, and ++++ callus exceeding the surface of the agarose bead

**Figure 2.**
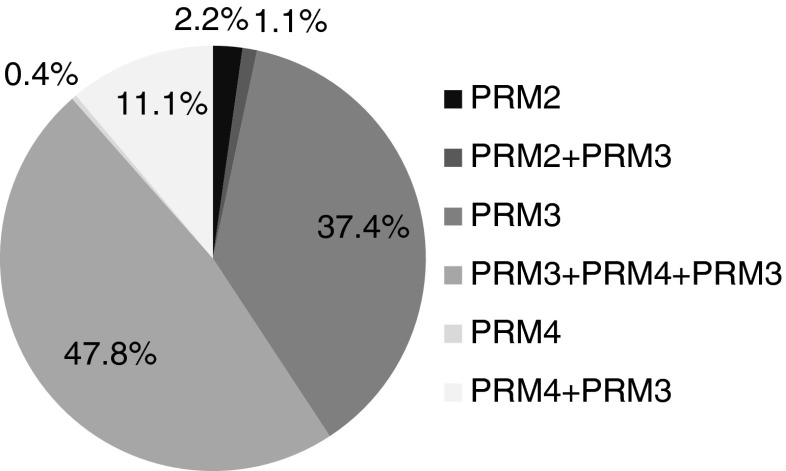
Percentages of *G. decumbens* plants regenerated on specific PRM media and media combinations.

Studies that have described plant regeneration from gentian mesophyll protoplasts indicate that the induction of morphogenesis is probably an intrinsic bottleneck in the process (Jomori *et al.* 1995; Takahata *et al.* 1995). Only Nakano *et al.* (1995) obtained a high frequency of shoot organogenesis (up to 28.6%) for some genotypes of *G. triflora* and *G. triflora × G. scabra*, using high concentrations of TDZ (5–10 mg l^−1^) in combination with 0.1 mg l^−1^ NAA in the regeneration medium. As emphasized by Takahata *et al.* (1995), TDZ was expected to be exploited for tissue and protoplast culture of a wide range of *Gentiana* species. However, in the present experiments, after transferring protoplast-derived calli of *G. decumbens* onto plant regeneration media, no shoots regenerated on PRM1 medium, which contained 0.1 mg l^−1^ NAA and 8 mg l^−1^ TDZ (Table 4). In contrast, the use of PRM3 medium, which ensured induction of somatic embryogenesis from callus and cell suspensions of *Gentiana pannonica*, *G. cruciata*, *G. tibetica* (Mikuła and Rybczyński 2001; Mikuła *et al.* 2002), and *G. kurroo* (Fiuk and Rybczyński 2008a) and from protoplasts of *G. kurroo* cell suspensions (Fiuk and Rybczyński 2007), led to somatic embryos formation from protoplast-derived tissues of *G. decumbens*. The highest number of embryos per agarose bead (2.5) was obtained from tissues cultured previously on CPM3 medium. Fewer somatic embryos (1.0 and 0.17) were regenerated from calli formed on CPM4 and CPM2 media, respectively. Somatic embryogenesis was also induced on other PRM media, but with lower efficiency. A repetitive embryogenic response of callus cells was observed on PRM2 medium after proliferation on CPM4 medium, with just over two embryos produced per agarose bead. On PRM4 medium, which facilitated somatic embryogenesis in protoplast cultures of *G. crassicaulis* (Meng *et al.* 1996), embryos were produced only from callus formed on CPM4 medium. It is noteworthy that sequential culture on PRM4 followed by PRM3, or on PRM3 followed by PRM4 and again on PRM3, increased the number of plants finally obtained over those obtained on PRM4 or PRM3, respectively (Fig. 2). Out of over 50 regenerants in total, 97.4% were obtained with the use of PRM3 medium alone or in combination with another medium. The highest percentage of plants (47.8%) came from tissues cultured sequentially on PRM3, PRM4, and PRM3 media.

**Table 4.** Number of somatic embryos per agarose bead (100 μl) regenerated after 8 wk on different plant regeneration media (PRM)

Callus proliferation medium	Plant regeneration medium			
	PRM1	PRM2	PRM3	PRM4
CPM1	0 b	0.50 ± 0.50 ab	0 b	0 b
CPM2	0 b	0 b	0.17 ± 0.29 ab	0 b
CPM3	0 b	0 b	2.50 ± 2.29 a	0 b
CPM4	0 b	2.17 ± 1.04 ab	1.00 ± 1.00 ab	1.17 ± 1.26 ab

Values followed by the same *letter* are not significantly different at *P* < 0.05 (HSD test)

Most of the mature somatic embryos (Fig. 1*h*) easily converted into plantlets on half-strength MS medium with 2% (*w/v*) sucrose. Regenerated plants (Fig. 1*i*) grew vigorously on full-strength MS medium. Apart from leaf blades (Fig. 1*j*) that were wider than those of parent plants (Fig. 1*k*), regenerants showed no visible morphological differences under *in vitro* conditions.

### Nuclear DNA Content, Number of Chromosomes, and Stomatal Characteristics.

Cytological and cytogenetic analysis of regenerated plants is an indispensable element of research on protoplast culture systems, since the conditions of protoplast isolation and culture, as well as indirect plant regeneration through callus, can induce somaclonal variation, which is often manifested as changed ploidy (Nassour *et al.* 2003; Sheng *et al.* 2011). Indeed, within the family Gentianaceae, tetraploid plants were regenerated from protoplasts of shoot tips of *E. grandiflorum* (Lindsay *et al.* 1994), and tetraploids and hexaploids were regenerated from cell suspension-derived protoplasts of *G. kurroo* (Fiuk and Rybczyński 2007).

**Figure 3.**
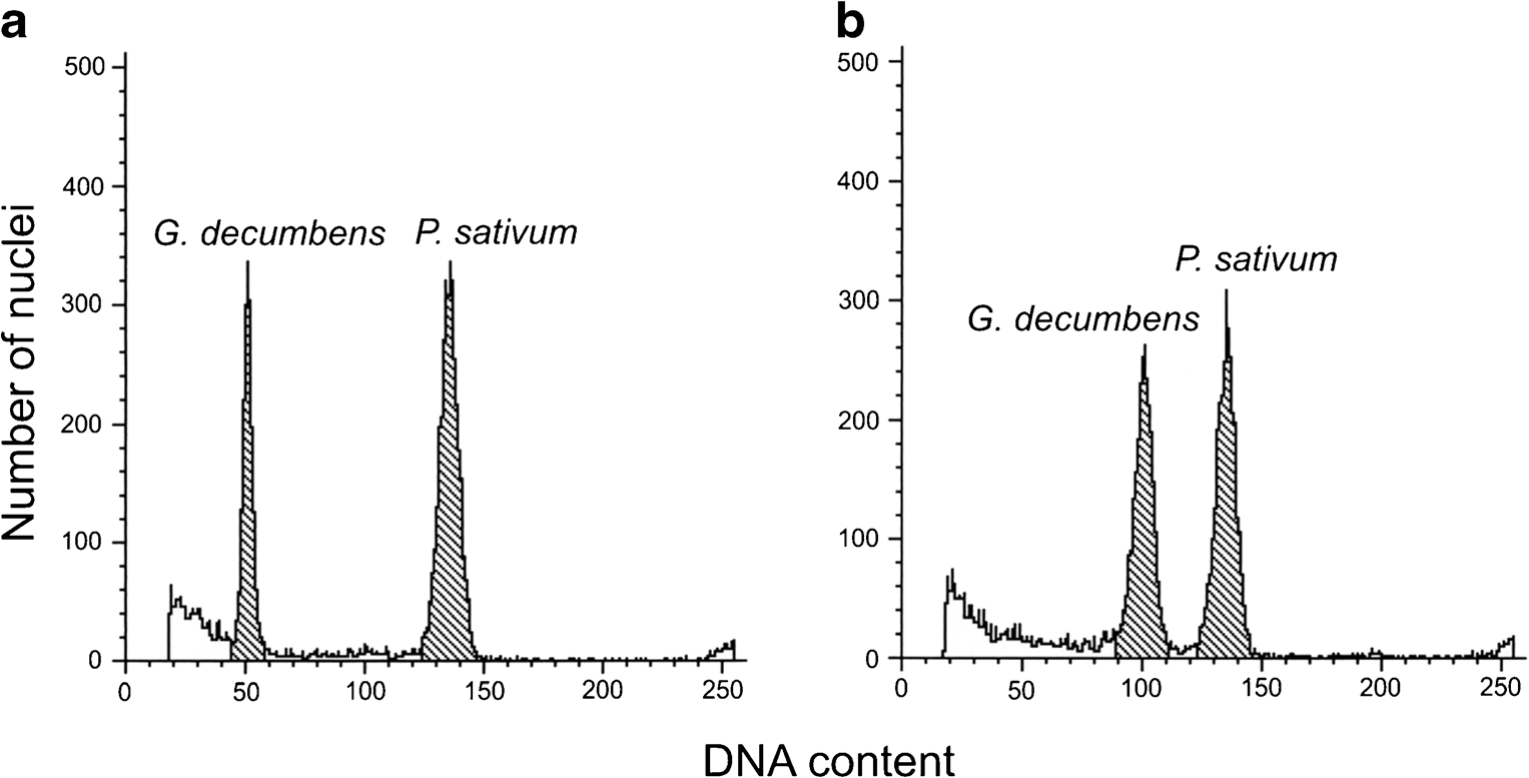
DNA histograms of nuclei isolated simultaneously from leaves of *Pisum sativum* (internal standard) and *Gentiana decumbens*. (*a*) Seed-derived control plant. (*b*) protoplast-derived regenerant.

**Figure 4.**
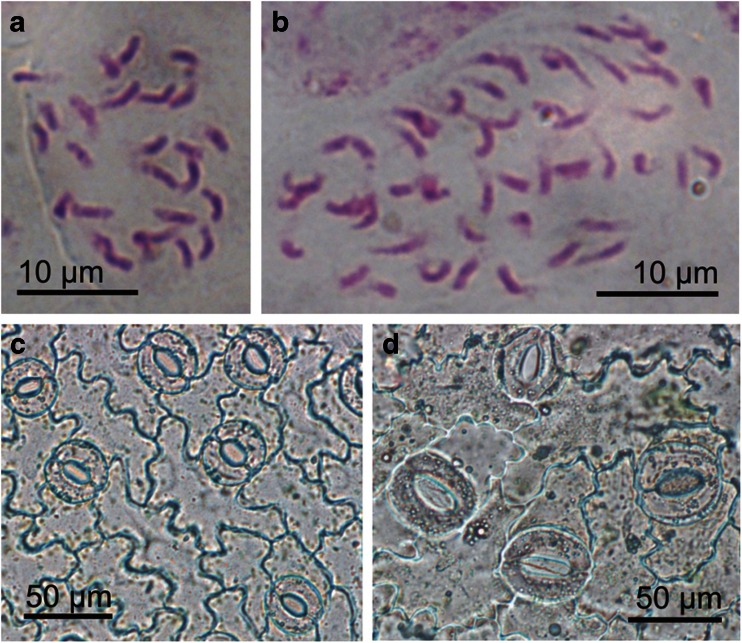
Chromosome counting and stomatal characteristics of *G. decumbens*. (*a*, *b*) Mitotic metaphase chromosomes in root-tip cells of a seed-derived parent plant (*a*) and a protoplast-derived regenerant (*b*). (*c*, *d*) Stomata of a seed-derived parent plant (*c*) and a tetraploid protoplast-derived regenerant (*d*).

Flow cytometry is a fast and accurate method of estimating of nuclear DNA content, widely used for analysis of plant material cultured *in vitro* (Ochatt *et al.* 2000; Nassour *et al.* 2003; Thiem and Sliwinska 2003; Elmaghrabi and Ochatt 2006; Konieczny *et al.* 2012). Its use for the evaluation of *G. decumbens* tissue cultures revealed that the diploid, seed-derived *in vitro*-grown plants had 3.49 pg/2C DNA (Fig. 3*a* and Table 5). A similar genome size (3.63 pg/2C) for *in vivo*-grown plants of this species was reported by Mishiba *et al.* (2009). However, all 21 protoplast-derived regenerants tested had approximately twofold the DNA content of the seed-derived plants (Fig. 3*b*) and thus were tetraploid. The results of flow cytometry were confirmed by chromosome counting. In root-tip cells of plants regenerated from protoplasts, 52 chromosomes were present (Fig. 4*b*), which was twice the number in root-tip cells of control plants (Fig. 4*a* and Table 5).

**Table 5.** Cytological characteristics of *G. decumbens* seed-derived plants (control) and plants regenerated from leaf mesophyll protoplasts

Plant type	2C DNA content (pg)	Number of chromosomes	Stomata length (μm)	Stomata width (μm)	Number of stomata per square millimeter of leaf blade area
Control	3.49 ± 0.07 b	26	35.99 ± 3.05 b	33.84 ± 6.26 b	189.73 ± 44.98 a
Regenerant	6.84 ± 0.18 a	52	50.55 ± 4.04 a	44.17 ± 5.43 a	89.41 ± 26.04 b

Values in the same *column* followed by the same *letter* are not significantly different at *P* < 0.05 (HSD test)

Chromosome doubling in plants often results in various morphological and anatomical changes (Dhooghe *et al.* 2011). Tetraploid *Eustoma* plants had a range of physical features such as increased width and length of the leaves and bigger flowers (Lindsay *et al.* 1994). Tetraploid plants of *G. triflora* were also easily distinguishable from diploids by their increased leaf thickness (Morgan *et al.* 2003). As seed- and protoplast-derived plants of *G. decumbens* showed no conspicuous morphological differences *in vitro*, except in leaf width, additional indirect markers of polyploidy were tested in order to check their use in distinguishing tetraploid plants from diploids. Measurements of stomata and counting of chloroplasts are easy to perform and less laborious than chromosome counting (Sari *et al.* 1999). Moreover, in contrast to flow cytometry, their application does not require expensive equipment. The present study showed that stomatal characteristics could be used to evaluate ploidy in *G. decumbens*, since plants regenerated from protoplasts (4*x*) possessed stomata on their abaxial leaf surfaces that were significantly larger than stomata of parent plants (2*x*) growing under the same conditions (Fig. 4*c*, *d* and Table 5). The density of the stomata in regenerated tetraploid plants was about half that in control plants (Table 5).

It is unclear why 100% regenerated plants of *G. decumbens* were autotetraploid. The fully expanded, young gentian leaves used as a protoplast source contained almost exclusively nuclei with a 2C DNA content (Fig. 3*a*), so it is not likely that the donor tissue itself was the source of tetraploid cells, unless all of the 2C cells left the cell cycle and were arrested at the G_0_ state. Bergounioux *et al.* (1988), studying the ability of protoplasts that originated from cells at different phases of the cell cycle to undergo cell division, concluded that the differentiated G_0_ state is not conducive to division. However, in the tissue they used as a source of protoplasts, 4C cells (being at the G_2_ phase of the cell cycle) occurred at a higher frequency than in the leaves of *G. decumbens*. Another reason for regeneration of polyploid plants can be spontaneous fusions during protoplast isolation, which occur especially when protoplasts are prepared from actively dividing cultured cells (Bhojwani and Razdan 1996). However, exposing the donor cells to a strong plasmolyticum before enzyme treatment, as in this study, should sever the plasmodesmatal connection and, consequently, reduce the frequency of spontaneous fusion. Thus, it is more likely that divisions of protoplast-derived cells were disturbed. As reported previously, protoplast cultures are especially unstable because of a high frequency of errors in microtubule synthesis, spindle formation and orientation, and chromatid segregation (Karp 1994). Moreover, as reported for *Nicotiana plumbaginifolia* Viv. and *Brassica napus* L., protoplast-derived cells with a 4C nuclear DNA content can divide faster that those with a 2C DNA content (Magnien *et al.* 1982; Chen *et al.* 1994).

Yet another possibility is that polyploidization occurred during callus formation. As shown for *P. sativum* (Ochatt *et al.* 2000) and *Medicago truncatula* Gaertn. (Elmaghrabi and Ochatt 2006), callus cells often undergo endoreduplication (amplification of DNA without mitosis), which causes an increase in the frequency of cells with a DNA content higher than 2C. The combination of plant growth regulators added to the culture media could promote a certain pathway of DNA amplification (endoreduplication or cell division; Lukaszewska *et al.* 2012). Verification of the regeneration mechanism of tetraploid *G. decumbens* plants will require further cytogenetic studies. Nevertheless, the results presented here support the suggestion that cells with higher ploidy can reenter the cell cycle more easily than cells with 2C DNA content in *in vitro* culture (Magnien *et al.* 1982; Chen *et al.* 1994).

Although somaclonal variation is undesirable for micropropagation or *ex situ* conservation of plant diversity (Takagi *et al.* 2011), it can provide a useful means of plant genetic improvement (Karp 1994). Polyploidy can lead directly to morphological, anatomical, and physiological changes, which may be exploited in plant breeding for the production of cultivars with increased ornamental value or higher capacity to cope with different stresses (Dhooghe *et al.* 2011). For medicinal species, polyploids often exhibit enhanced production and/or qualitative improvement in the biochemical profile of active compounds (Dhawan and Lavania 1996). Thus, further assessment of the morphological traits of autotetraploid *G. decumbens* plants for their horticultural potential, as well as for analysis of secondary metabolites, is worthwhile.
